# Las conversaciones transatlánticas del doctor Honorio Delgado, 1930-1940

**DOI:** 10.1590/S0104-59702025000100059

**Published:** 2026-02-09

**Authors:** Irène Favier

**Affiliations:** i Directora, Institut Français d’Études Andines. Lima – Peru; ii Profesora investigadora, Laboratoire de Recherche Historique Rhône-Alpes/Université Grenoble Alpes. Grenoble – Francia. irene.favier@univ-grenoble-alpes.fr

**Keywords:** Historia de la salud, Honorio Delgado (1892-1969, Psiquiatría, Germanofilia, Tercer Reich, History of health, Honorio Delgado (1892-1969, Psychiatry, Germanophilia, Third Reich

## Abstract

La trayectoria de Honorio Delgado ocupa un lugar importante, aunque ambivalente, en la historia de la psiquiatría peruana y latinoamericana. Como introductor del psicoanálisis y luego feroz opositor del freudismo en nombre de un enfoque biomédico de los trastornos mentales, examinamos su viraje a partir de fuentes adicionales. Esta nota de investigación examina los viajes del psiquiatra a Alemania y los informes de sus libros, y plantea la hipótesis de que factores extracientíficos, relacionados con sus afinidades con el régimen nazi, también pueden haber contribuido a este rechazo. El arraigo transatlántico de Delgado en una conversación científica dominada por la Europa de la época puede haber influido en sus posiciones.

## Abstract (en)

The career of Honorio Delgado is a significant, albeit complex, position in the history of Peruvian and Latin American psychiatry. As an introducer of psychoanalysis and later a fierce opponent of Freudianism in the name of a biomedical approach to mental disorders, we examine his shift based on additional sources. This research note examines the psychiatrist’s journeys to Germany and the influence of his books. It also hypothesizes that extrascientific factors, related to his connections with the Nazi regime, may also have contributed to this rejection. Delgado’s transatlantic engagement in a scientific conversation dominated by Europe at the time may have influenced his positions.

Esta nota de investigación se propone abordar un capítulo de la trayectoria profesional intelectual del médico psiquiatra peruano Honorio Delgado (1892-1969), cuya figura conoció un proceso de “totemización” mediante un uso colectivo y profesional de la herramienta biográfica, que resultó en la edificación de una figura referencial, aunque a menudo también ambivalente. Delgado fue tanto el ferviente introductor del psicoanálisis en América latina a partir de 1915 como su principal detractor a partir de 1940 en beneficio de un enfoque biomédico, consagrado con la introducción de la clorpromazina en la caja de herramientas psiquiátrica en el Perú a partir de 1953.

A continuación, se presentan algunas incursiones en la vida de un pionero de la psiquiatría peruana, que se proponen participar de un afán colectivo más amplio en curso en el espacio académico iberoamericano. En efecto, un conjunto de estudios recientes ofrece una revisión de la trayectoria biográfica de algunas figuras fundacionales, alimentada por los aportes de la historia social, cultural, intelectual, de la ciencia o desde el enfoque de la salud pública (Huertas, 2014; Sacristán, 2007; Ruperthuz Honorato, 2014; Sosenski, Sosenski, 2010; Ramacciotti, 2018; Venancio, Franco Potengy, 2015; Marquieri, 2020; Mercier, Martins, 2023). Estos trabajos no solo contribuyeron a socavar el enfoque difusionista que la historiografía centrada en lo europeo brindaba a las dinámicas psiquiátricas del siglo XX, sino que también proponen una vía complementaria a narrativas de índole profesionalizante. A continuación, se abordan tres aspectos de la vida de Honorio Delgado que parecen esclarecedores en cuanto a su trayectoria intelectual: sus años de formación en Arequipa y luego en Lima; sus viajes a Europa; y sus lecturas intensivas y plurilingües, pertenecientes a la conversación eugenésica de las décadas de 1930 y 1940.

## Arequipa de fin de siglo

Tanto los años de socialización intelectual de Honorio Delgado como los de inicios de su desempeño laboral apuntan a una trayectoria que cumple con los requisitos de un *cursus honorum* tal como se concebía a inicios del siglo XX. Delgado provenía de una familia adinerada terrateniente de Arequipa, segunda ciudad del país cuyo papel, tanto económico como político, se había mantenido a lo largo de la época republicana, inclusive durante la guerra del Pacífico. Arequipa constituyó un centro de formación y del debate recurrente sobre las vías de la modernidad (McEvoy, 1997). Honorio Delgado socializa dentro de aquella élite, cuyas prácticas sociales aplica: se escolariza dentro del ámbito religioso en el colegio San Vicente de Paul, donde estudia francés y latín, luego en el prestigioso colegio Independencia hasta 1908. Empieza sus estudios superiores en Arequipa, donde obtuvo el bachiller en la Universidad de San Agustín. Su entorno social cercano comparte un apego a cierta continuidad cultural, heredada de la época colonial, y con ella una concepción de la sociedad peruana basada en el fundamento de la Iglesia católica.

Esta juventud en la Arequipa de fin de siglo trae consigo ciertos rasgos que parecen haber sido formativos, no solo en cuanto a su formación intelectual e ideológica, sino también en lo que respecta a su ulterior consagración narrativa mediante el uso del género biográfico, a través de diversos homenajes que se le rendieron. En efecto, Delgado frecuenta personalidades notables de su tiempo: es cercano a Víctor Andrés Belaunde, a quien conoció en 1919 gracias a Cristóbal de Losada y Puga, y con quien se reunían José Gálvez, José María de la Jara, Carlos Ledgard, José de la Riva Agüero, Guillermo Leguía y Mariano Ibérico. Dentro de este entorno, si bien divergen los posicionamientos – José Gálvez resultaría más liberal que Riva Agüero a lo largo de los años –, se nota cierta sintonía que llevó a designarlos como pertenecientes a la llamada “generación del 900 o generación arielista”, según el ensayo de José Enrique Rodó *Ariel* (publicado en 1900), donde se defiende un impulso colectivo por desacreditar el modelo norteamericano, al que se le iba reprochando su materialismo (Zariquiegui, 2023). Asimismo, la corriente arielista ponía hincapié en un supuesto genio artístico e identitario suramericano, del que se subrayaba una identidad concebida como más deseable por su aliento artístico: la elevación espiritual es concebida por el mismo Rodó, de otro lado, como reacia a la emergencia de un protagonismo de las llamadas masas en la toma de decisiones políticas. Son estos miembros de la generación del 900 quienes, más tarde, formularían homenajes a Delgado, a la hora de transfigurar su trayectoria en una serie de logros retrospectivos, tal como es el caso, en 1957, de Cristóbal Losada y Puga a la hora de celebrar su aniversario.

En buena medida, Honorio Delgado reproduce esta sociabilidad de élite en Lima, donde se traslada a inicios de los años 1910: ahí se construye su figura como referencia imprescindible en el intento de establecer un sistema de atención en salud mental. El apoyo de una clase social parece crucial en este punto: en Lima, si bien los años de juventud y de formación intelectual lo llevan a involucrarse en espacios de vanguardia, tal como la revista *Amauta*, su trayectoria y la de José Carlos Mariátegui rápidamente divergen (Johnson Lothrop, 2020), a tal punto que Delgado parece haber participado de lo que se podría plantear como una vanguardia conservadora, pues la modernidad concebida a partir de un enfoque conservador resulta ser una vertiente significativa de este pensamiento colectivo. A pesar de haber frecuentado grupos de distintas orientaciones, y de que la generación del 900 conformara más un “mosaico intelectual” (Zariquiegui, 2023) que una entidad ideológicamente homogénea, se notó una tendencia al conservatismo dentro de los ambientes que Delgado escogió como próximos. A lo largo de su vida intelectual, mantuvo un afán por participar de una modernidad en construcción en el siglo XX, como lo sugieren sus numerosas contribuciones periodísticas, en particular en el diario *El Comercio*. Esta contribución al debate público sobre qué trayectoria escoger para un Perú “moderno” ha sido permanente y su producción escrita, tanto mediática como académica, apunta a referencias ideológicas conservadoras, apegadas a un orden social, a menudo prerrepublicano.

Entre Arequipa y Lima, la socialización de Honorio Delgado se realizó mediante circulaciones intranacionales que desempeñaron un rol notable en la formación de vínculos de amistad duraderos, que resultarán cruciales a la hora de elaborar una narrativa dedicada a la consagración biográfica de Delgado como pionero de la medicina. Otras circulaciones parecen haber tenido un rol fundamental en la trayectoria, en buena medida triunfal, de Honorio Delgado. Se trata de las que ocurrieron a escalas regional e internacional, que nos proponemos examinar a continuación.

## Algunos puntos ciegos de esta trayectoria transatlántica

En un ensayo reciente, en buena medida programático, Marcos Cueto (2013) delinea algunas pistas para recurrir a la biografía, entre las que figura la historia mundial. En el caso de Honorio Delgado, los viajes a Europa invitan a considerar la dimensión internacional de la trayectoria del psiquiatra, cuya legitimidad se nutrió de sus conexiones más allá del Perú, a donde precisamente traía novedades e innovaciones. Si bien la germanofilia de Delgado es conocida, estos viajes a Europa en la década de 1930 han sido poco comentados por la literatura médica. En cuanto a la historiografía, esta sí ha subrayado la importancia de las circulaciones en la conformación de este itinerario intelectual, profesional y hasta ideológico, perteneciente a la construcción colectiva, mundial y plural de la eugenesia, un movimiento cuyo propósito fue mejorar la supuesta raza nacional mediante una serie de medidas variadas según los contextos, desde la prevención por canales pedagógicos hasta la violencia estatal (Rey de Castro, 1993; Stucchi-Portocarrero, 2018; Ríos Molina, 2023). La lectura de una serie de fuentes extranjeras y la relectura de algunas fuentes endógenas al campo psiquiátrico peruano sugieren profundizar el contraste entre estas dos tradiciones historiográficas.

Para ello, este texto propone examinar dos actividades importantes a la hora de propiciar la internalización del campo psiquiátrico: los viajes y las lecturas plurilingües realizadas por Delgado. Estas prácticas de intercambios profesionales resultaron clave, ya que la psiquiatría se encontraba en pleno proceso de institucionalización a principios del siglo XX, y se trataba tanto de encontrar aliados como, según sugiero interpretar, de participar de una conversación parcialmente mundial con los agentes de los principales polos de referencias médicas: Estados Unidos y, sobre todo, Europa, Francia y Alemania en el caso de Delgado. Sin embargo, este proceso de socialización internacional se desconoce parcialmente en los textos panegíricos tradicionales: si bien se suele mencionar el periodo de vinculación de Delgado con Freud a fines de la década de 1910, o su giro farmacológico decisivo mediante la introducción de la clorpromazina en la década de 1950 (Cruzado, 2013), existen algunos puntos ciegos que merecen ser examinados. No solo se trata de entender el espectacular vuelco de posicionamiento de Delgado – discípulo de Freud, cuyo enfoque psicoanalítico primero introdujo en América Latina en 1915, luego actor de su “enjuiciamiento” –, sino también de comprender bajo qué mecanismos funcionan los espacios médicos a lo largo del siglo XX, y qué relaciones se establecieron entre los centros de producción de conocimiento y sus peripecias.

## Los viajes de Honorio: fuentes escasas, pero que llaman la atención

Los viajes de Honorio Delgado constituyen un primer punto ciego, que puede merecer una indagación archivística que permita revisar o tal vez matizar el retrato que se le suele hacer, además de completar el estudio de viajes hechos en el sentido contrario, como en el caso de médicos desplazándose hacia los Andes (Lossio, 2006). A partir de la década de 1920, empieza a desplazarse por Europa, según una dinámica germanocéntrica. En noviembre de 1922, está en Weimar, Alemania, para el séptimo Congreso Internacional de Psicoanálisis, al que finalmente no logra asistir por un retraso del barco. En 1925, el entonces presidente Augusto Leguía le otorga el estatuto de representante del Gobierno peruano en Alemania, donde asiste al séptimo Congreso Mundial de Psicoanálisis (PAAA, 13 mar. 1923); en 1924-1925, se casa con una ciudadana alemana, Helene Rehe (1901-1983), de quien al parecer existen muy pocas fuentes accesibles (algo bastante común, lamentablemente), con la excepción del archivo Delgado que alberga la Pontificia Universidad Católica del Perú, donde se documenta su esfuerzo epistolar por conservar la documentación de su esposo (Rehe, 1971-1982). En 1927, logra asistir al décimo Congreso de Psicoanálisis en Innsbruck, en Austria, y aprovecha esta oportunidad para visitar a Freud en su casa de Semmering. Delgado ya había establecido una relación epistolar con el psicoanalista desde 1919, que duraría hasta 1934, cuando empieza su distanciamiento progresivo pero contundente. Un año después de la muerte de Freud, en 1940, Delgado expresa de forma explícita este escepticismo, lo que se torna pronto en un rechazo, un posicionamiento relativamente nuevo a pesar de nunca haber sido un freudiano ortodoxo. La historiografía a menudo se ha preguntado cómo entender esta evolución bastante ortogonal. Si bien los textos publicados en el campo profesional han subrayado la discrepancia existente entre el giro biologista y farmacológico de Delgado en la década de 1950 y el apego mantenido hacia el psicoanálisis de, Carlos Alberto Seguín, su sobrino, poco se ha escrito sobre las décadas de 1930 y 1940, cuando se manifiesta por primera vez este distanciamiento.

Una mirada a los archivos alemanes de este periodo sugiere una serie de posibles motivos extracientíficos a este giro científico: así, la historia de las ciencias ha subrayado con frecuencia los vínculos entre estas últimas y el contexto en que se producen. Los archivos del Ministerio Alemán de Asuntos Exteriores ponen de relieve un vínculo continuo entre Honorio Delgado y las instancias alemanas. Así, en 1935, las autoridades alemanas lo identifican como un simpatizante (PAAA, 2 ago. 1935): “El Dr. García-Frías, director del sanatorio para enfermos pulmonares de Jauja, es nombrado médico proalemán. Estudió en Alemania. De los médicos de Lima, se mencionan como médicos con actitud favorable hacia nosotros: el Dr. Mauricio Dávila, el Dr. Honorio Delgado, el Dr. Augusto Dammert, el Dr. Mackormack (ciudadano de los Estados Unidos)”.

En 1940, Honorio Delgado recibe la “Medalla por los Servicios prestados a la Amistad y a la Ciencia” por parte del Instituto Iberoamericano de Berlín, conocido por sus vínculos con el régimen nazi (PAAA, ene.-mayo 1961; Quiénes somos, 2024); en 1949, funda la Asociación Cultural Peruano Alemana (PAAA, ene.-mayo 1961); en 1953, se dirige hacia las autoridades diplomáticas alemanas en Lima para proponer que el modelo alemán sirva de inspiración en el Perú (PAAA, 24 ago. 1953); en 1961, reciba la cruz del mérito (PAAA, ene.-mayo 1961). Se le presenta como “uno de los pocos académicos peruanos conocidos más allá de las fronteras del Perú”. En base a su participación en la Academia de Medicina Germano-Iberoamericana como miembro honorario y a la entrega en 1940 de la “Medalla por Servicios a Amistad y a la Ciencia” del Instituto Iberoamericano de Berlín, se le propone la entrega de tal condecoración como “un justo homenaje y reconocimiento a su labor en favor de los intereses alemanes”. Esta continuidad del vínculo con Alemania no deja de interrogarnos: ¿se trata de un apego más allá de la Segunda Guerra Mundial, o fue este periodo clave del régimen nazi un momento especial en la formación del posicionamiento del doctor Delgado?

## Leer en tiempos tormentosos: volver a fuentes tradicionales de la historia de la psiquiatría

Tal interrogante invita a revisar fuentes más tradicionales de la historia de la psiquiatría, es decir, las revistas, a pesar de que las corrientes actuales de esta historia invitan a buscar más allá de ellas, en un afán por diversificar las fuentes. Sin embargo, llaman la atención particularmente algunos textos que conforman estas revistas, sobre todo la *Revista de Neuropsiquiatría*, que fundaron Honorio Delgado y Julio Óscar Trelles en 1938 (Alva, 2015). Esta fecha es clave según la hipótesis seguida por esta nota de investigación, por lo que las numerosas reseñas que acompañan los artículos, y que suelen generar un interés historiográfico menor, merecen ser leídas. Ahí se encuentran observaciones curiosas por su poca cientificidad, que sugieren, nuevamente, tanto la probable influencia del contexto tan tenso de la década de 1930, como el deseo profundo de quienes participaban de la “excelencia en la periferia” (Cueto, 1989) por superar la relegación geopolítica que padecían en el campo científico.

La preocupación de Delgado por dar cuenta de las recientes publicaciones parece perceptible, al punto de incurrir en algunas imprecisiones. Así, en 1938, ofrece una revisión del libro *Race, hérédité, folie*, del eugenista francés antisemita René Martial, uno de los mayores expertos en selección racial bajo el régimen de Vichy (Noiriel, 2007). Presentándose como un “estudio de antroposociología aplicado a la inmigración”, el libro se centra en la prevalencia de la locura entre los judíos y sus presuntas estadísticas en Francia. Refleja las preocupaciones biologizantes del periodo francés de entreguerras y la construcción intelectual de un supuesto problema racial y de su contraparte, la construcción de una solución a este problema preconstruido: la de la profilaxis médica eugenésica, incluida dentro de la especialidad psiquiátrica. El libro inspira a Delgado a realizar una asombrosa comparación numérica con la población judía supuestamente alojada en el Hospital Víctor Larco Herrera: “En este estudio, el doctor Martial expone sus preocupaciones y los resultados de su investigación sobre el mestizaje, con especial referencia al pueblo francés. Es una llamada de atención, complementada en otro tono por la sensacional obra de Céline, Bagatelles pour un massacre, publicada el año pasado” (Delgado, 1938).

La articulación entre mestizaje, inmigración y locura llaman la atención de Honorio Delgado, quien parece vislumbrar una posible conexión con el contexto peruano. Cita el texto de R. Martial por su pretensión estadística:

Puesto que el mestizaje sigue inevitablemente a la inmigración, … hay que … hacer una selección estricta cuando se trata de la mezcla de pueblos … La frecuencia de la locura entre los inmigrantes es un hecho bien conocido, que también se ha comprobado en Francia, aunque las referencias no son muy precisas debido a la falta de controles fronterizos. Es importante señalar que un tercio de los enfermos mentales extranjeros internados en los manicomios del Sena pertenecen a la raza asiática … En Polonia y Suiza, la población judía está dos veces más alienada que la no judía (Delgado, 1938).

Luego establece un paralelo en cifras con la realidad peruana de la cual se presenta como el testigo privilegiado. En efecto, puntúa la cita con un llamativo comentario entre paréntesis: “(Un dato digno de mención es que en nuestro departamento de HVLH, los judíos representan hoy el 10 % de la población total y el 66 % de la población extranjera)” (Delgado, 1938).

Estas cifras no se logran corroborar con los registros de ingreso del hospital (lo que sugiere la pertinencia de una ampliación de las fuentes disponibles en torno a la historia de la psiquiatría): una mirada siquiera rápida a estas fuentes revela la abrumadora mayoría de sujetos católicos que ingresaron en el hospital (Delgado, 1938). ¿Qué lleva a Delgado a incluir este comentario? Una hipótesis podría ser este deseo de participar de una conversación mundial, lo que lo llevaría a recurrir al lenguaje antisemita en boga en aquella época, a costa de la veracidad de los hechos.

En 1940, Honorio Delgado se hace eco de un número dirigido por Carl Schneider para la revista *Archiv für Psychiatrie und Nervenkrankenheiten*, titulado “Arte degenerado y arte de los alienados”, cuyas conclusiones alaba y profesa compartir (Delgado, 1940), a pesar de que llevaba veinte años desarrollando un innovador taller de arte-terapia, que había dirigido César Moro y que lo llevó a intercambiar ideas sobre esa práctica con Hans Prinzhorn, quien desarrolló en Alemania una importante colección de arte psicopatológico, y quien fue perseguido por el Tercer Reich:

No cabe comparar la mentalidad del sujeto pseudoartista propenso al arte degenerado con el verdadero talento o genio creador. No hay continuidad entre las manifestaciones de un niño o de un negro, un alienado, un cubista o dadaísta y un verdadero artífice … Ahí está el elemento común entre el arte de los alienados y el arte degenerado (dadaísta, futurista, expresionista etc., etc.), con sus extremos esquizofrénico y judeo-comunista (Delgado, 1940).

En 1941, Honorio Delgado reprocha a Theodore Reik, cuya obra *Thirty years with Freud* reseña su supuesto espíritu de resentimiento cuando evoca el trato reservado a Freud por el régimen nazi (Delgado, 1941): “En realidad, es un libro de contenido muy poco orgánico y con un través de muy mal gusto: de principio a fin insinúa el odio a los estadistas europeos que han expulsado a los judíos de sus respectivos países. El lector tiene a cada momento la impresión de que el autor no es un psicólogo objetivo, sino que ‘respira por la herida’”.

Elogia el trabajo realizado en Alemania desde 1927 por el profesor Walter Jaensch en medicina constitucional (Delgado, 1942), quien desde el 1 de abril de 1940 era director de un nuevo instituto universitario y policlínico en la Beneficencia de Berlín y miembro del Partido Nacionalsocialista desde 1933: “Dos medidas de las que cabe esperar buenos resultados son la educación desde la tierna infancia en el pensamiento del bien de la comunidad y el estímulo de la alegría en el trabajo, cuya aplicación ha dado ya frutos apreciables en Alemania, gracias al perfeccionamiento de las instituciones encargadas de ponerlas en practica” (Delgado, 1942).

Abundan ejemplos de este tipo, que como mínimo confirman la prevalencia del paradigma eugenista en la psiquiatría peruana (Ríos, 2023; Stucchi-Portocarrero, 2018). Mas allá quizás, y desde una perspectiva biográfica, estos ejemplos también sugieren aventurarse en los archivos alemanes que, si bien no parecen hasta ahora documentar su círculo matrimonial ni sus viajes a congresos, no guardan silencio tampoco entorno a sus simpatías ideológicas. Dan cuenta también, muy probablemente, de un afán de participar de una modernidad, monopolizada por centros ubicados fuera de América Latina, sin que el costo de tal participación sea siempre importante.

## Consideraciones finales

Una indagación en las fuentes alemanas permite contribuir a la conversación historiográfica sobre el motivo del giro delgadiano de lo psicoanalítico a lo biológico en cuanto al debate sobre la etiología de las enfermedades mentales, formulado de forma tenue poco antes de la muerte de Freud, luego de manera más estruendosa a inicios de la compleja década de 1940. Asimismo, la atención prestada a las lecturas de Honorio Delgado permite formular una hipótesis sobre los mecanismos que lo llevaron a dedicarse a la corriente eugenista: esta última puede haberse alimentado del doble contexto de su socialización temprana y de su construcción como figura intelectual a escala regional e internacional. Una serie de consideraciones biográficas contextualizadas sugieren que su participación activa en la eugenesia puede deberse a un cierto antijudaísmo originado en su educación temprana.

¿Se trata de una mera germanofilia o de un apoyo directo al régimen nazi? Lo segundo parece definitivamente haberse nutrido de lo primero. Si bien las fuentes no son suficientes en esta etapa para afirmar un posicionamiento explícito y público a favor del régimen vigente en Alemania en aquella época, tres factores inclinan hacia ambas opciones. Primero, las fuentes demuestran un afán de la diplomacia nazi por vincularse con intelectuales latinoamericanos, de acuerdo a las líneas definidas por la Conferencia Latinoamericana en Berlín del 9 de junio de 1939 (Lorini, 2016). Segundo, este interés de Delgado por el Tercer Reich también participa de un afán existente en el Perú por vincularse e inspirarse del proyecto fascista que se iba armando en aquella época (Villarías Robles, 1997). Tercero, puede haberse alimentado también de una inquietud por desarrollar una cercanía con los centros de producción académica de la época, cuando Alemania y Francia beneficiaban todavía de cierta centralidad intelectual. Este eurocentrismo explicaría la posición de Delgado en las décadas de 1960 y 1970 frente al auge del interés por herramientas terapéuticas provenientes de los Andes por parte de la psiquiatría peruana e inclusive por alumnos suyos como Javier Mariátegui. Estos se volvieron promotores de la psiquiatría comunitaria en aquella época, y desarrollaron una dinámica científica más centrada en lo nacional y lo regional, de cuyo desarrollo no pudo ser testigo tras su fallecimiento en 1969, pero cuya génesis desaprobó.

Así pues, más allá de la innegable y atractiva eficiencia lograda en la década de 1950 por el enfoque biofarmacéutico hacia el tratamiento del sufrimiento mental, la cronología sugiere que los factores extracientíficos también pueden haber desempañado un rol en los posicionamientos de este médico en las décadas de 1930 y 1940, años que fueron cruciales a nivel mundial.
